# The F-Box Protein TaFBA1 Positively Regulates Drought Resistance and Yield Traits in Wheat

**DOI:** 10.3390/plants13182588

**Published:** 2024-09-16

**Authors:** Qinxue Li, Xiaoyu Zhao, Jiajie Wu, Huixia Shou, Wei Wang

**Affiliations:** 1 The Provincial International Science and Technology Cooperation Base on Engineering Biology, International Campus of Zhejiang University, Haining 314400, China; chen979402575@126.com; 2 National Key Laboratory of Wheat Improvement, Shandong Agricultural University, Tai’an 271018, China; zxyaworld95@163.com (X.Z.); jiajiewu@sdau.edu.cn (J.W.)

**Keywords:** wheat (*Triticum aestivum* L.), F-box protein, drought tolerance, antioxidant capacity, water absorption

## Abstract

Environmental stresses, including drought stress, seriously threaten food security. Previous studies reported that wheat F-box protein, TaFBA1, responds to abiotic stresses in tobacco. Here, we generated transgenic wheat with enhanced (overexpression, OE) or suppressed (RNA interference, RNAi) expression of *TaFBA1*. The *TaFBA1*-OE seedlings showed enhanced drought tolerance, as measured by survival rate and fresh weight under severe drought stress, whereas the RNAi plants showed the opposite phenotype. Furthermore, the OE plants had stronger antioxidant capacity compared to WT and RNAi plants and maintained stomatal opening, which resulted in higher water loss under drought stress. However, stronger water absorption capacity in OE roots contributed to higher relative water contents in leaves under drought stress. Moreover, the postponed stomatal closure in OE lines helped to maintain photosynthesis machinery to produce more photoassimilate and ultimately larger seed size. Transcriptomic analyses conducted on WT and OE plants showed that genes involved in antioxidant, fatty acid and lipid metabolism and cellulose synthesis were significantly induced by drought stress in the leaves of OE lines. Together, our studies determined that the F-box protein TaFBA1 modulated drought tolerance and affected yield in wheat and the *TaFBA1* gene could provide a desirable target for further breeding of wheat.

## 1. Introduction

As a food crop that is a major source of starch and calories throughout the world, wheat (*Triticum aestivum* L.) plays an important role in food security in many countries, including China [1]. Although wheat yields have significantly increased due to the breeding of new varieties in recent years, extreme environmental conditions, like drought, are still major factors limiting production [2,3,4]. Therefore, identifying the key genes and molecular mechanisms that participate in the response to drought is of great significance to maintaining high yields of this essential crop under adverse conditions. There have been more and more studies reporting that some genes are involved in wheat drought tolerance [5,6]. Qiu et al. [7] showed that overexpression of TaASR1-D in wheat could improve osmotic and drought tolerance by affecting reactive oxygen species (ROS) accumulation and ABA signaling. Overexpressing ABA receptor TaPYL1-1B increased the water-use efficiency of transgenic wheat by regulating ABA and drought response genes under drought conditions [8]. Moreover, the wheat DREB transcription factor TaDTG6-B also functioned as a positive regulation factor of wheat drought tolerance [9]. All of these studies on wheat drought tolerance provide important theoretical bases for breeding wheat to handle various abiotic stresses, especially drought.

Extreme and unpredictable environments usually result in severe damage to plants, which cannot move to escape outside stresses [10]. To adapt to stress conditions, plants can only react at the molecular and biochemical levels, such as rapidly decreasing undesirable proteins and increasing protective functions [11]. The ubiquitin-26S proteasome system (UPS) utilizes three types of enzymes, Ubiquitin (Ub)-activating enzymes (E1s), Ub-conjugating enzymes (E2s), and Ub ligases (E3s), to rapidly and effectively select intracellular proteins for degradation. E3s recognize the target proteins and label them for later degradation, and many E3s are reported to be involved in abiotic stress response in plants [12,13]. In wheat, overexpression of the U-box E3 ligase TaPUB1 enhances salt stress tolerance through interacting with α-mannosidase protein TaMP [12]. The SKP1/CUL1/F-box (SCF) complex is the best-characterized class of E3 ligases, among which the F-box-containing protein is responsible for recognizing the substrates and initiating responses to abiotic stresses [13]. Researchers have reported various roles of F-box proteins during abiotic stresses by different regulation mechanisms. In *Arabidopsis*, the auxin-mediated stress response factor AtFBA1, an F-box protein, conferred tolerance to salt and osmotic stress by triggering an ABA-mediated plant response [14]. And F-box protein AtPP2-B11 influences the expression of Na^+^ homeostasis genes under salt stress, and AtPP2-B11-OE lines exhibited lower Na^+^ accumulation in *Arabidopsis* [15]. Moreover, Sharma et al. [16] showed that *OsFBX257*, a rice F-box protein-coding gene, influenced leaf and grain length, and number of panicles, while significantly increasing the grain yield under drought stress. However, some F-box proteins function in regulating plant abiotic stress negatively. For example, luciferase and Yeast-2-Hybrid (Y2H) assay revealed that GhTULP34, a protein containing the F-box domain, interacted with GhSKP1A, suggesting its negative role in osmotic stress regulation [17]. Zhang et al. reported that the overexpression of DOR1 led to increased drought sensitivity, indicating that DOR1 acted as a negative regulator of drought stress tolerance [18]. 

Wheat is a crop mainly grown in arid and semi-arid areas, where it can easily be subject to drought, which leads to severe yield losses [2]. Despite an increasing number of genes recently reported to regulate drought tolerance in wheat, only a small number of E3 ligases are known to be involved in wheat drought response, with almost all either U-box or Ring finger E3 ligases [3,7]. As an important member of the SCF complexes, F-box proteins play important roles in response to abiotic and biotic stresses in plants [19,20]. Genes including the F-box domain have been identified in many species of plants, including *Arabidopsis*, tobacco, rice, and wheat. However, there is limited research available on F-box proteins in wheat exposed to abiotic stresses, especially drought stress [21,22]. TaFBA1 is an F-box protein and a previous study reported that it improved the drought and salt stress tolerance of tobacco by increasing the antioxidant ability and maximizing intracellular Na^+^ compartmentalization, respectively [23,24]. An et al. [6] first indicated that TaFBA1-overexpressing *Arabidopsis* was insensitive to ABA and TaFBA1 regulation on drought tolerance may be independent of ABA synthesis. Given that *TaFBA1* is a gene from wheat. In this study, the drought tolerance of wheat was investigated by overexpressing and repressing the F-box-encoding gene *TaFBA1*. The results demonstrated that the overexpression of *TaFBA1* significantly enhanced drought tolerance and affected grain yield in wheat. These findings provide new insights into the roles of TaFBA1 in drought tolerance and its potential application in the improvement of abiotic stress resistance in wheat.

## 2. Results

### 2.1. Generation and Identification of TaFBA1-Overexpressing and TaFBA1-RNAi Wheat Lines

**Figure 1:**
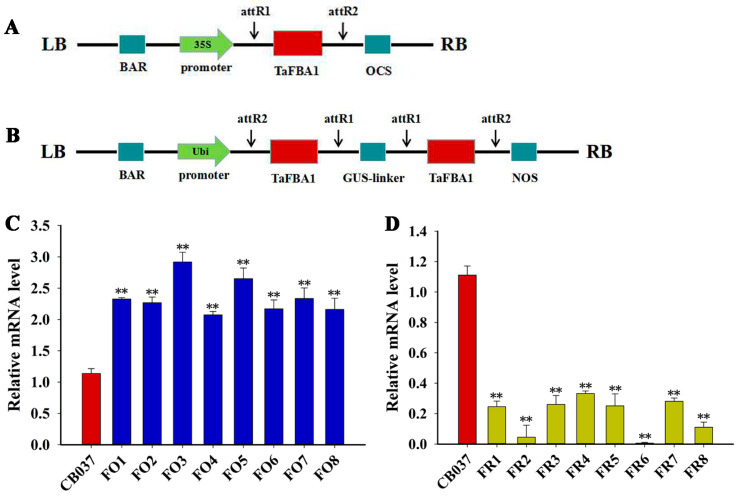
Molecular identification of TaFBA1 transgenic lines. Schematic diagrams of constructs used for (**A**) TaFBA1 overexpression (FO) and (**B**) RNAi-mediated knockdown (FR). The expression levels of TaFBA1 in (**C**) FO and (**D**) FR lines were assessed using qRT-PCR. The data represent the mean ± SD of three biological replicates. ** *p* < 0.01.

The previous works showed that the wheat F-box gene *TaFBA1* could improve plant tolerance to several abiotic stresses, including salt, drought, and heat in transgenic tobacco [13,23,24]. To explore the biological functions of *TaFBA1* in wheat, the cultivar CB037 (wild type) was used for transformation and transgenic lines either overexpressing or knocking down *TaFBA1* were generated (Figure 1A,B). We obtained more than 50 *TaFBA1* RNA interference (*TaFBA1*-RNAi) lines and 80 *TaFBA1*-overexpression (*TaFBA1*-OE) lines. Quantitative real-time PCR (qRT-PCR) analysis confirmed that the transcription of *TaFBA1* was reduced in the RNAi lines (FR) and increased in the overexpression lines (FO; Figure 1C,D). Two overexpression lines (FO3, FO5) and knockdown lines (FR2, FR8) were chosen for the next experiments. E3 ligase activities were examined in CB037 and transgenic lines (FO and FR) under normal and drought conditions. The results showed that the OE lines had higher E3 ligase activity than that of WT with or without drought stress, while the RNAi lines exhibited the inverse trend. All the lines showed higher enzyme activities after drought treatment (Figure S1). These results indicated that *TaFBA1* was successfully overexpressed or silenced, both transcriptionally and translationally, in the transgenic wheat plants.

### 2.2. TaFBA1 Overexpression Confers Drought Tolerance to Wheat at Seedling and Heading Stages

To dissect the function of *TaFBA1* in wheat drought tolerance, the phenotype of WT, FO3 and FR2 seedlings were photographed, and the OE plants showed significantly better growth status than WT and RNAi plants. Since there are many differences between growth in hydroponics and soil, the responses of all the lines were examined to drought stress in a potting mix and obtained similar phenotypes (Figure S2B). At this stage, no morphological or developmental abnormalities were apparent in any of the lines under normal conditions. However, the OE lines showed greater tolerance to the drought stress (Figure S2B). After dehydration for 25 days, the OE lines were slightly wilted, while over 50% of leaves in the RNAi lines were severely wilted and ~41% were wilted in WT (Figure S2C). With continuous growth for 28 days, the fresh weight of all seedlings did increase, even under drought stress, but the fresh weight of OE lines was significantly higher than those of WT, while that of RNAi lines and WT were quite similar (Figure S2D). These results indicated that the OE lines were less sensitive to drought compared with WT and RNAi plants and that *TaFBA1* overexpression could significantly improve drought tolerance in wheat at the seedling stage.

**Figure 2:**
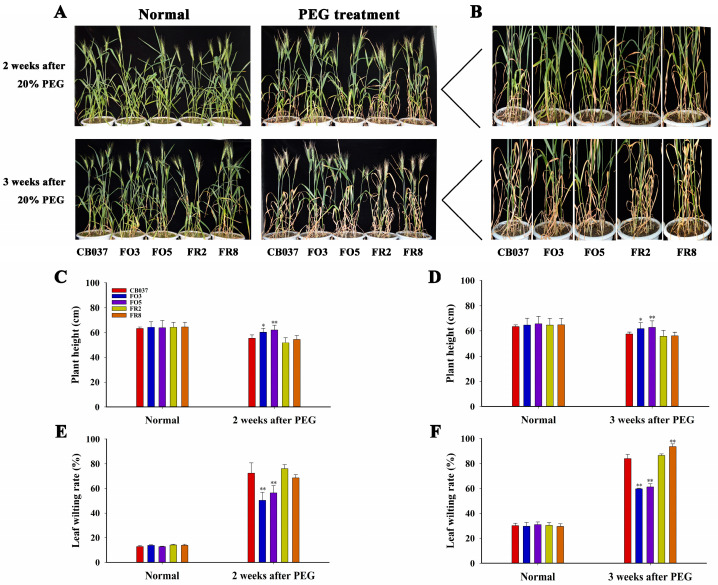
Analysis of drought tolerance in WT and *TaFBA1* transgenic wheat lines at the heading stage. (**A**) Phenotype and (**B**) magnifying local picture of 32-day-old wheat exposed to 20% PEG6000 for 2 and 3 weeks. Plant height after drought stress for (**C**) 2 weeks and (**D**) 3 weeks, respectively. Leaf wilting rate (%) after drought stress for (**E**) 2 weeks and (**F**) 3 weeks. The data represent the mean ± SD of three biological replicates. * *p* < 0.05; ** *p* < 0.01.

For mature plants, all genotypes showed similar growth under well-watered conditions, and there was no difference in plant height. However, when exposed to 20% PEG6000, signs of stress were less prominent in the OE lines after being exposed to drought stress for 2 or 3 weeks, with fewer leaf-wilting symptoms and taller plants. However, the RNAi lines showed a higher rate of leaf wilting and were shorter (Figure 2). The above results suggested the overexpression of *TaFBA1* could confer drought tolerance at both the wheat seedling and heading stages.

### 2.3. TaFBA1 Overexpression Impacted Spike Weight and Grain Size

**Figure 3:**
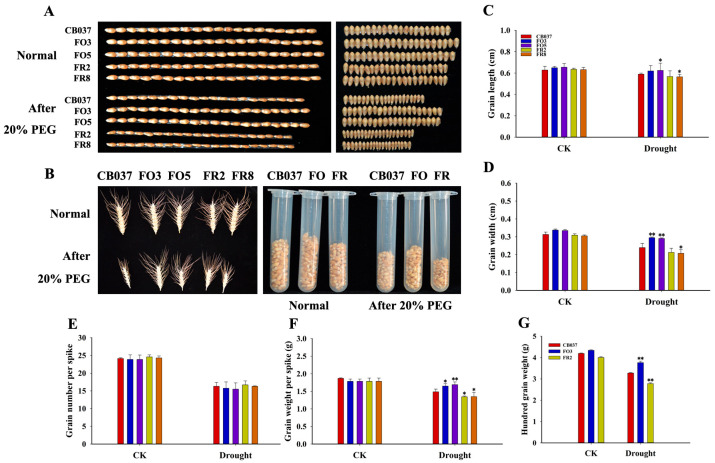
Effects of *TaFBA1* overexpression on wheat grain traits under normal and drought stress conditions. (**A**) Grain length and width phenotype of 20 mature wheat grains harvested under normal and 20% PEG6000 treatment conditions. (**B**) Spike size and hundred-grain volume of mature wheat grains. (**C**) Average grain length. (**D**) Average grain width. (**E**) Grain number per spike. (**F**) Grain weight per spike. (**G**) Hundred-grain weight. The data represent the mean ± SD of three biological replicates. * *p* < 0.05; ** *p* < 0.01.

Next, it was observed that yield-related traits of the WT, *TaFBA1*-OE and *TaFBA1*-RNAi lines under normal and dehydration conditions. As shown in Figure 3, under normal growth condition, the grains of the OE lines were a little longer and wider than WT (Figure 3A,C,D), causing a slight increase in the 100-grain volume and weight (Figure 3B(right),G) compared with WT. The grains of the RNAi lines were slightly thinner than WT, while the grain length was comparable to WT (Figure 3A–D,G). The differences in grain size among the lines were even more striking after drought treatment (Figure 3A–D). Examination of grain weight per spike revealed a significant loss in the WT and RNAi lines but not the OE lines (Figure 3F), while the number of grains per spike was similar between all lines under either normal or dehydration conditions (Figure 3E). The above results suggested that *TaFBA1* played positive roles in the yield traits of wheat, especially under drought conditions.

### 2.4. TaFBA1 Overexpression Improved the Photosynthetic Capacity of Transgenic Wheat under Drought Stress

**Figure 4:**
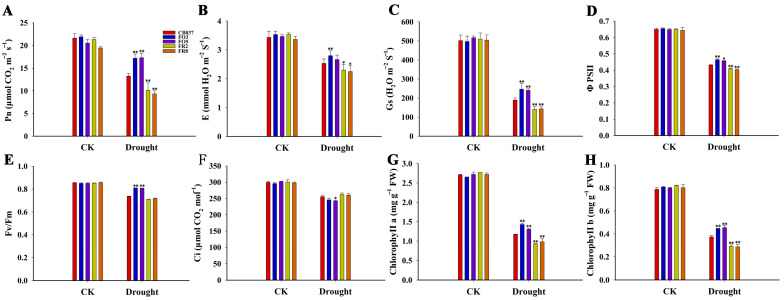
Photosynthetic capacity of WT and transgenic wheat lines under drought stress. WT, *TaFBA1*-OE and *TaFBA1*-RNAi wheat lines were grown under normal condition and under dehydration with 20% PEG6000 for 2 weeks before determination of the (**A**) Net photosynthetic rate (Pn), the (**B**) transpiration rate (E), the (**C**) stomatal conductance (Gs), the (**D**) actual PSII efficiency (ΦPSII), the (**E**) maximum photochemical efficiency of PSII (Fv/Fm), the (F) intercellular CO_2_ concentration (Ci), the contents of (**G**) chlorophyll a and (**H**) chlorophyll b. The data represent the mean ± SD of three biological replicates. * *p* < 0.05; ** *p* < 0.01.

To evaluate the effects of drought on plant photosynthetic activity, the flag leaves of all the lines were used to assess eight photosynthetic parameters. Under control conditions, there were no obvious differences in these photosynthetic parameters between the transgenic and WT plants. After PEG treatment, the net photosynthesis (Pn) rate decreased in all lines, although the Pn rate of the OE lines was still higher than WT and RNAi lines (Figure 4A). The variations among the transpiration rate (E, Figure 4B) and stomatal conductance (Gs, Figure 4C) were consistent with those of the Pn values. Similar results were also observed for photosystem II photochemical potential (Fv/Fm) and quantum yield of electron transfer through PSII (ΦPSII), but the lines exhibited minor differences (Figure 4D,E). The intercellular CO_2_ concentration (Ci) was similar in all lines, although the value was slightly lower in the OE lines, opposite the effect on the Pn rate (Figure 4F). The chlorophyll content often reflects the degree of leaf chlorosis [25,26]. We found that the contents of chlorophyll a and chlorophyll b were reduced by an average of 45% and 43% in OE lines, respectively, but by 56% and 54% in WT and 67% and 63% in RNAi plants after dehydration stress (Figure 4G,H). These results further demonstrated that *TaFBA1* played positive roles in improving drought tolerance by supporting photosynthetic capacity in wheat.

### 2.5. TaFBA1 Overexpression Alleviated Oxidative Damage in Wheat under Drought Stress

**Figure 5:**
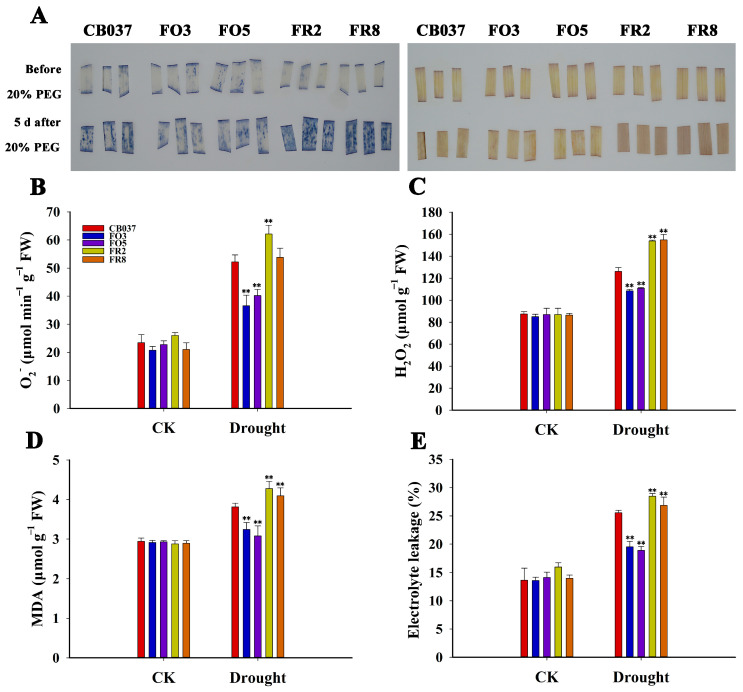
Reactive Oxygen Species (ROS) accumulation and cell membrane oxidative damage in WT and transgenic lines under drought stress. Two-week-old seedlings of WT, *TaFBA1*-OE and *TaFBA1*-RNAi wheat exposed to PEG6000 as dehydration stress for about 5 days. Leaves were stained with (**A**) NBT and (**B**) DAB staining for detecting O^2−^ and H_2_O_2_ levels. Quantification of (**B**) O^2−^ and (**C**) H_2_O_2_ levels in leaves as above. The (**D**) MDA content and (**E**) relative electrolyte leakage were determined in the lines grown in normal and dehydration conditions. Protein carbonylation, as another measure of oxidative stress is presented in Figure S3. The data represent the mean ± SD of three biological replicates. ** *p* < 0.01.

NBT and DAB staining are often used as indicators of ROS (mainly O^2−^ and H_2_O_2_) accumulation. Under well-watered conditions, there was no significant difference in nitroblue tetrazolium (NBT) and 3,3′-diaminobenzidine (DAB) staining between WT and transgenic plants. After drought treatment, the staining was deepest in the leaves of the RNAi lines, while that of the OE lines was the lightest (Figure 5A). Quantitation of the O^2−^ and H_2_O_2_ levels were consistent with the staining results (Figure 5B,C). This indicated that *TaFBA1*-OE plants accumulated less ROS under drought stress. Moreover, following PEG treatment for 5 days, the protein carbonylation level of the OE lines was less than the WT and RNAi lines (Figure S3). This indicated that the proteins suffered less damage from oxidation under drought stress. 

The level of malondialdehyde (MDA) is an indicator of oxidation of lipids, and oxidation of lipids can lead to disruption of the cell membrane, which can be measured as electrolyte leakage. From Figure 5D,E, the drought-treated *TaFBA1*-OE lines had significantly lower MDA contents and less electrolyte leakage relative to the WT, while the RNAi lines showed an opposite trend, which suggested that the cell membranes in the *TaFBA1*-RNAi lines are more severely damaged by ROS. 

### 2.6. TaFBA1 Overexpression Enhanced the ROS Scavenging Capacity of Wheat under Drought Stress

**Figure 6:**
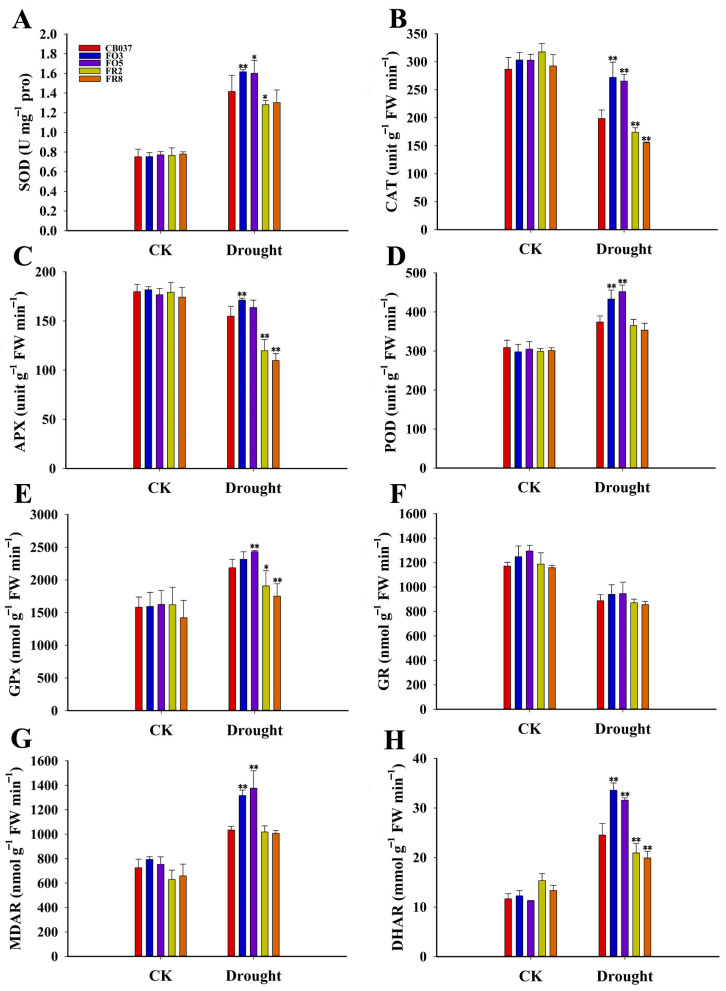
Antioxidative abilities of WT and transgenic wheat lines under normal and drought stress. Two-week-old seedlings of WT, *TaFBA1*-OE and *TaFBA1*-RNAi wheat exposed to PEG6000 as dehydration stress for about 5 days. Leaves were sampled for the determination of antioxidase enzyme activities. SOD (**A**), CAT, (**B**) APX (**C**), POD (**D**), GPx (**E**), GR (**F**), MDAR (**G**), and DHAR (**H**) activities in wheat grown under normal and drought stress conditions. The data represent the mean ± SD of three biological replicates. * *p* <0.05; ** *p*< 0.01.

To determine whether ROS were detoxified more rapidly in the OE lines or whether plants had a stronger metabolic capacity to cope with elevated ROS, the activities of several key antioxidase, including super oxide dismutase (SOD), catalase (CAT), ascorbate peroxidase (APX), peroxidase (POD), and glutathione peroxidase (GPx) were examined in leaves exposed to drought or PEG for 5 days (Figure 6). After drought stress, the SOD, POD and GPx activities increased in all lines, with higher values in the OE lines than WT and RNAi lines (Figure 6A,D,E). Although the levels of both CAT and APX activity decreased after drought stress, the values were still higher in the OE lines (Figure 6B,C). These results suggested that the *TaFBA1*-OE lines were more effective in terms of ROS detoxification than the WT and RNAi lines.

The same approach was taken regarding the activities of enzymes in the ascorbate (AsA)-GSH cycle, including monodehydroascorbate reductase (MDAR), dehydroascorbate reductase (DHAR), and glutathione reductase (GR). The activity of GR was slightly decreased after drought treatment, but there was no significant difference between the lines (Figure 6F). The MDAR and DHAR activities were up-regulated significantly after dehydration, with a greater increase in the OE lines than WT and RNAi lines (Figure 6G,H). Together, these results suggested that *TaFBA1* promoted the activity of the enzymes in the AsA-GSH cycle, which are involved in cellular redox homeostasis.

**Figure 7:**
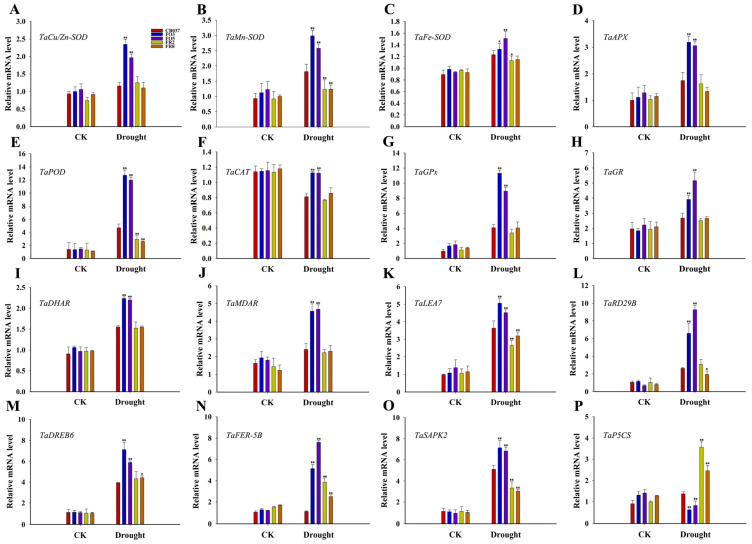
Relative expression of antioxidant-related and stress-responsive genes in WT and transgenic wheat lines. Relative expression of antioxidant-related genes, namely *TaCu/Zn-SOD* (**A**), *TaMn-SOD* (**B**), *TaFe-SOD* (**C**), *TaAPX* (**D**), *TaPOD* (**E**), *TaCAT* (**F**), and *TaGPX* (**G**), *TaGR* (**H**), *TaDHAR* (**I**), and *TaMDAR* (**J**) and stress-responsive genes, namely *TaLEA7* (**K**), *TaRD29B* (**L**), *TaDREB6* (**M**), *TaFER-5B* (**N**), *TaSAPK2* (**O**), and *TaP5CS* (**P**), in the flag leaves of WT and transgenic wheat under drought stress. The data represent the mean ± SD of three biological replicates. * *p* < 0.05; ** *p* < 0.01.

The transcripts of some antioxidation-related genes, such as *TaCu/Zn-SOD*, *TaMn-SOD*, *TaFe-SOD*, *TaCAT*, *TaAPX*, *TaPOD*, *TaGPx*, *TaDHAR* and *TaMDAR* were monitored in the flag leaves of plants exposed to PEG (Figure 7). The results showed that the expression trends of the most genes discussed above were consistent with their enzyme activities, with the exception of *TaAPX*, whose expression level was increased in all lines after dehydration. However, different from the GR activity (Figure 6F), the expression of *TaGR* increased in all lines after PEG treatment and had significantly higher levels in the OE lines (Figure 7H). Collectively, the gene transcripts showed a greater increase in the OE lines than WT and RNAi lines under drought stress. Additionally, we determined the transcript levels of several stress-related genes, including, *TaLEA7* (late embryo genesis abundant protein), *TaRD29B* (responsive to desiccation 29B), *TaDREB6* (dehydration responsive element binding protein6), *TaFER-5B* (Ferritin), *TaSAPK2* (sucrose non-fermenting1-type Ser/Thr protein kinase), and *TaP5CS* (Delta 1-pyrroline-5-carboxylate synthetase) (Figure 7K–P). There were no notable differences in transcript levels among all the lines under normal conditions. Five of these stress-responsive genes, *TaLEA7*, *TaRD29B*, *TaDREB6*, *TaFER-5B*, and *TaSAPK2*, were up-regulated in all lines when plants were exposed to drought stress, but the transcript levels increased to a larger extent in the OE lines compared to the WT. These results suggested that the higher drought tolerance observed in the OE lines might result from increased transcript levels of stress-related and antioxidant-related genes, which would reduce the ROS content due to the enhancement of antioxidant capability by *TaFBA1* overexpression. However, the expression level of *TaP5CS*, which encodes a proline synthesis or catabolism enzyme, was the lowest in the OE lines (Figure 7P). This result is consistent with the trend of proline content in Figure 10G. 

Further, methyl viologen (MV) was sprayed on the wheat leaves as an external oxidative stress. Fourteen days after treatment with MV, the OE lines exhibited less leaf wilting and higher total Chl contents than that of the WT and RNAi lines, which suggested that the overexpression of *TaFBA1* significantly increased the antioxidant capacity of transgenic wheat under drought stress (Figure S4).

### 2.7. TaFBA1 Overexpression Supported Stomatal Opening

**Figure 8:**
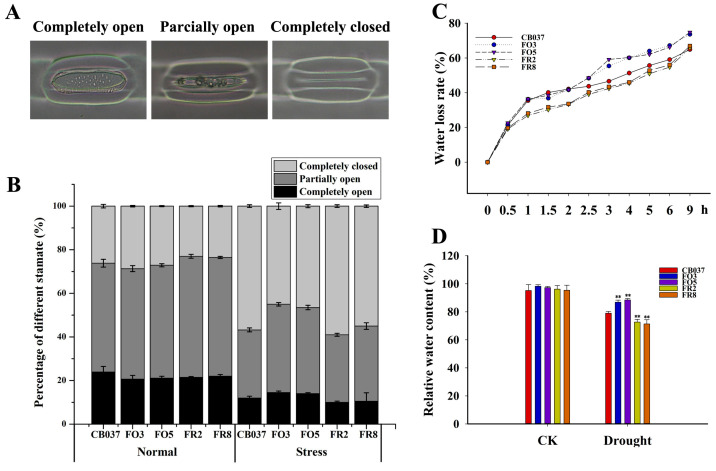
Stomatal aperture on the surface of WT and transgenic wheat lines leaves. (**A**) Images of stoma with different aperture on the leaves of WT and transgenic wheat after PEG treatment obtained using a fluorescence microscope. (**B**) Percentage of stoma of different aperture. (**C**) Water loss rate of detached leaves of WT and transgenic wheat lines. (**D**) Relative water content (RWC) in leaves of all lines grown under normal and drought stress conditions. The data represent the mean ± SD of three biological replicates. ** *p* < 0.01.

To verify whether the drought-tolerant phenotype of OE lines was derived from better water maintenance capacity, the stomatal aperture was first categorized as three levels (completely open, partially open, and completely closed) in the WT, OE and RNAi lines under normal and drought conditions via microscopy. Following drought stress, 45% of the stoma were completely closed in the OE lines but 56% were closed in WT, while 15% were completely open in the OE lines while only 12% were completely open in WT. The percentage of partially open stoma was 40% and 32% in OE lines and WT, respectively (Figure 8A,B). Moreover, the water loss rates of detached leaves showed that the TaFBA1-OE lines lost water much more rapidly than WT (Figure 8C). 

**Figure 9:**
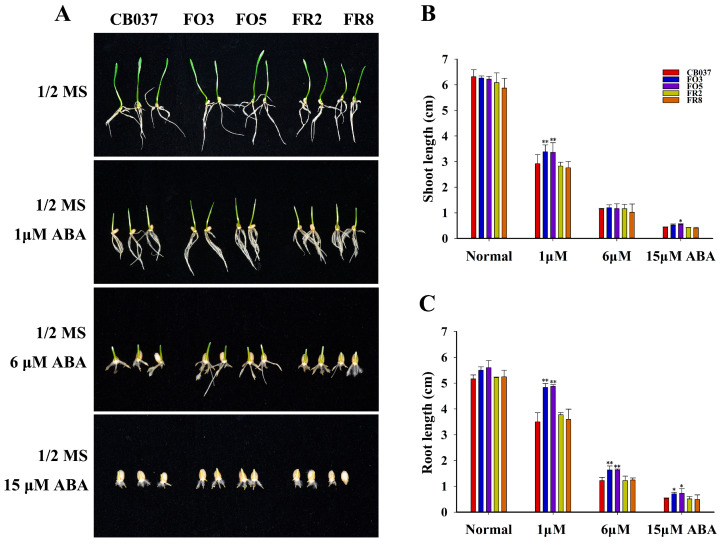
Response of shoot and root growth in germinating WT and transgenic wheat lines to ABA treatment. The (**A**) growth phenotype, (**B**) shoot length, and (**C**) primary root length of wheat seedlings germinated in the presence of different concentrations of ABA for 5 days. The data represent the mean ± SD of three biological replicates. * *p* < 0.05; ** *p* < 0.01.

The sensitivity of the wheat lines to ABA was determined using germinating seeds. As shown in Figure 9, the shoot length and root length of the OE lines were distinctly greater than the WT and RNAi lines under 1 μM ABA. With increasing ABA concentration, seedling growth of all lines was further limited, although root growth of the OE lines remained significantly greater than the WT and RNAi lines. This lack of ABA inhibition during germination indicated that *TaFBA1*-OE plants were less sensitive to ABA. 

### 2.8. TaFBA1 Overexpression Enhanced Root Water Absorption Capacity of Wheat under Drought Stress

**Figure 10:**
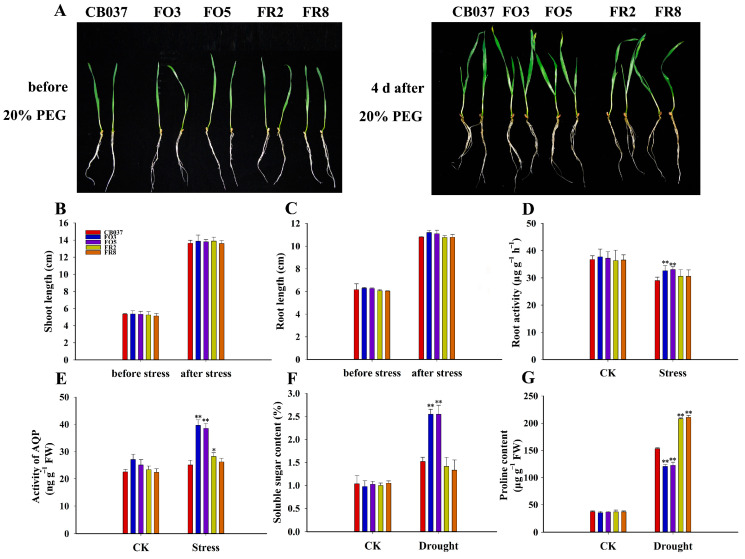
Effects of drought stress on root growth, water absorption ability, and osmotic adjustment substances contents in WT and transgenic wheat lines. The (**A**) root growth phenotype, (**B**) shoot length, and (**C**) root length of wheat seedlings before and after 20% PEG6000 treatment. (**D**) Root vitality and (**E**) AQP activity of wheat seedlings with and without 20% PEG6000 treatment for 4 days. The contents of (**F**) soluble sugar (**G**) and proline in all lines under drought stress for 5 days in soil mixture. The data represent the mean ± SD of three biological replicates. * *p* < 0.05; ** *p* < 0.01.

The root system is the main organ of the plant that absorbs water from soil [27,28]. To explore whether the increased drought tolerance of the OE lines was related to their root development, we observed the root architecture and counted the root length of all lines. The results showed that there was no significant difference in root growth status of all the lines before or after PEG treatment (Figure 10A–C). Surprisingly, the root vitality and aquaporin (AQP) activity of the OE lines were higher than that of WT after dehydration treatment (Figure 10D,E). It was speculated that the higher root vitality and AQP activity under drought stress enhanced the water absorption capacity of the OE lines, which could compensate for the water loss caused by the large stomatal opening while maintaining a high relative water content (RWC) under drought condition (Figure 8D).

Further, we determined the proline and soluble sugar contents in wheat, as these compounds are considered important metabolite contributors to osmotic adjustment [29]. The results showed that under normal conditions, both of soluble sugars and proline were at similar levels in all genotypes. After drought treatment, the contents of proline and soluble sugar increased in all lines, but the OE lines accumulated more soluble sugar than the WT and RNAi lines. However, the proline content showed an opposite trend after drought stress, with lower levels in the OE lines and higher levels in the RNAi lines (Figure 10F,G). These results suggested that it was soluble sugar but not proline that contributed to the increased drought tolerance of the OE lines. Overall, these observations supported the idea that *TaFBA1* could reduce wheat sensitivity to drought by increasing water absorption capacity and the accumulation of osmoprotectants.

### 2.9. Transcriptomic Analyses of CB037 and TaFBA1-OE Revealed Functions of TaFBA1 in Drought Tolerance

To gain insight into the molecular mechanisms underlying the *TaFBA1*-mediated response to drought stress, RNA sequencing was conducted on the FO3 and CB037 plants under normal and drought conditions. Before RNA sequencing, we tested the expression pattern of *TaFBA1* in leaves of CB037 grown in soil in response to 20% PEG6000 treatment. The results showed that *TaFBA1* was induced by drought stress, reaching peak expression at 6 h, and then decreasing over the next 18 h (Figure S5). This suggested that *TaFBA1* fairly functions early in the response to drought stress. 

**Figure 11:**
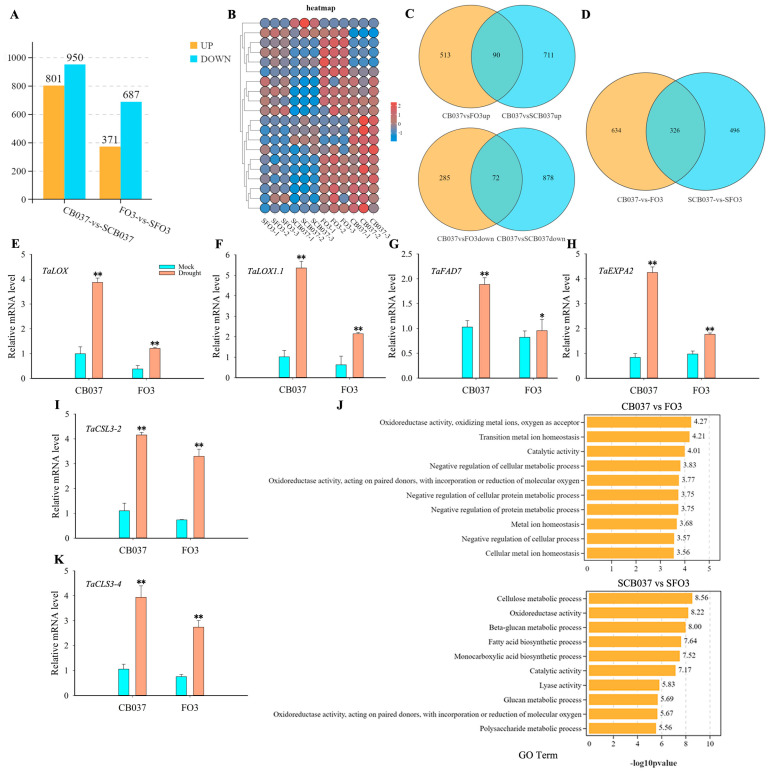
Transcriptome analysis of *TaFBA1*-OE wheat with or without drought treatment. (**A**) The number of up-regulated and down-regulated genes and (**B**) heatmap of expression profiles of DEGs in CB037 and SCB037 and in FO3 and SFO3. These comparisons are between plants of the same genotype grown under normal and dehydration conditions. (**C**) Venn diagrams showing the DEGs between different comparisons; CB037 vs. FO3up and CB037 vs. FO3down mean genes up-regulated and down-regulated in the well-watered OE line (FO3) compared to the well-watered WT (CB037); CB037 vs. SCB037up and CB037 vs. SCB037down mean genes up-regulated and down-regulated in dehydrated CB037 compared to well-watered CB037. (**D**) The DEGs in CB037 and FO3, SCB037 and SFO3. Relative expression levels of (**E**) *TaLOX*, (**F**) *TaLOX1.1*, (**G**) *TaFAD7*, (**H**) *TaEXPA2*, (**I**) *TaCSL3-2*, and (**K**) *TaCSL3-4*. (**J**) Top 10 enriched terms among the DEGs identified from CB037/FO3 and SCB037/SFO3, respectively. The data represent the mean ± SD of three biological replicates. * *p* < 0.05; ** *p* < 0.01.

Based on the expression pattern of *TaFBA1*, we stressed wheat plants with a 20% PEG6000 soil drench for 6 h (SCB037; SFO3) in parallel to growing plants without stress treatment (CB037; FO3) before sampling leaves for RNA extraction and sequencing. After the 6-h PEG6000 treatment, we identified 1751 differentially expressed genes (DEGs) between CB037 and SCB037 (Figure 11A) and constructed a heatmap visualizing the expression profiles of these DEGs (Figure 11B). However, PEG6000 treatment caused a less dramatic transcriptomic change in FO3 plants relative to CB037 (Figure 11A,B). These data suggested that *TaFBA1*-OE lines were less sensitive in terms of transcriptomic changes to drought stress than WT.

Venn diagram analyses indicated that 90 up-regulated genes were common between the comparisons of *TaFBA1*-OE to WT under the well-watered condition and of dehydrated versus well-watered WT plants, while 72 genes were shared between the down-regulated genes in these two comparisons (Figure 11C). These genes were differentially expressed not only in response to drought stress in CB037 but also in FO3 compared with the CB037 under normal conditions. These overlapping DEGs might have primed the *TaFBA1*-OE lines to better respond to drought stress, correlating to the stronger drought tolerance phenotype (Figure 2 and Figure S2). In addition, 326 DEGs were shared between the FO3/CB037 and SFO3/SCB037 comparisons (Figure 11D), suggesting that these genes were stably regulated by *TaFBA1* under both normal and drought conditions. 

The enrichment analysis was performed to classify the DEGs from the unstressed (FO3/CB037) and stressed (SFO3/SCB037) comparisons into various functional pathways based on gene ontology (GO). The top 10 enriched terms from each pairwise comparison were selected for further analyses (Figure 11J). Both the FO3/CB037 and SFO3/SCB037 comparisons included the terms “oxidoreductase activity” and “catalytic activity”. However, the DEGs from the SFO3/SCB037 comparison contained genes involved in the “fatty acid biosynthetic process” and “cellulose metabolic process”, while the FO3/CB037 comparison did not. This suggested that under normal conditions, *TaFBA1* mainly influences metal ion homeostasis and oxidoreductase activity, whereas under drought stress *TaFBA1* may affect drought tolerance through the regulation of oxidoreductase activity, fatty acid synthesis and lipid metabolism, as well as cellulose and beta-glucan metabolism. 

To further validate the transcriptomic data, six DEGs were selected among the genes included in the GO terms “oxidoreductase activity”, “fatty acid biosynthetic process” and “cellulose metabolic process”, namely *TaLOX* (lipoxygenase), *TaLOX1.1* (lipoxygenase 1.1), *TaFAD7* (omega-3 fatty acid desaturase 7), *TaEXPA2* (expansin-A2-like), *TaCSL3-2*, (mixed-linked glucan synthase 3-2), and *TaCSL3-4* (mixed-linked glucan synthase 3-4), for analysis by qRT-PCR. The results showed that these abiotic stress response genes were significantly up-regulated after drought treatment. Interestingly, the fold change of up-regulation was less in CB037 than in FO3 (Figure 11E–I,K). Meanwhile, the consistent results between this microarray data and qRT-PCR data above also confirmed the reliability of the transcriptome data in this paper. 

## 3. Discussion

### 3.1. TaFBA1 Positively Regulated the Drought Tolerance of Wheat

As global warming accelerates, droughts are likely to be more frequent and longer lasting. Wheat is a crop with a high risk of exposure to drought due to the geographical regions in which it is cultivated [2]. In order to survive under adverse environments, plants have evolved sophisticated mechanisms, including the degradation of proteins by UPS [13,30]. We transformed *TaFBA1* into wheat to generate overexpression and RNAi lines, which we used to explore *TaFBA1* response to drought (Figure 1). As shown in Figure 2 and Figure S2, all the *TaFBA1*-OE lines showed enhanced drought tolerance at both the seedling and heading stages. The photosynthetic system is usually susceptible to damage induced by drought. The effects are either direct, such as diffusion limitations through the stoma and the mesophyll and alterations in photosynthetic metabolism, or secondary, such as oxidative stress arising from the superimposition of multiple stresses [25]. In this study, the stronger photosynthetic capacity of the OE lines under drought stress implied greater production of photoassimilates, which may be an important factor in the bigger grains (Figure 3 and Figure 4). The results above suggested that TaFBA1 positively regulated drought tolerance in wheat. 

### 3.2. TaFBA1 Improved Drought Tolerance of Wheat by Enhanced Antioxidant Ability and Stress-Related Genes Regulation

Under drought stress, plants easily produce ROS, and excessive accumulation of ROS will cause oxidative stress, inhibit plant growth, and even cause cell death, so the ROS scavenging system plays an important role in reducing the harmful effects of drought stress [31,32]. Here, we found that the activities of some antioxidant enzymes were significantly higher in the OE lines than in WT and RNAi lines after drought stress (Figure 6), implying that these antioxidant enzymes played a major role in ROS clearance in OE lines. Plants respond to drought by regulating the transcription of corresponding genes [33]. In our research, many antioxidant-related and stress-related genes affected by *TaFBA1* also functioned in regulating the tolerance of *TaFBA1*-OE wheat (Figure 7). However, the expression trend of *TaP5CS* (Figure 7P) and the proline content (Figure 10G) in the OE lines indicated that proline is not the main permeable substance in TaFBA1-OE lines under drought stress. 

From the RNA-Seq analysis, we also found that genes related to cellulose metabolism, fatty acid synthesis and lipid metabolism showed significantly differential expression when the wheat seedlings were exposed to dehydration stress (Figure 11J). The plant cell wall plays an important role in the flexibility and stability of cell structures during plant development, while some cellulose synthesis-related genes regulate plant tolerance to abiotic stress [34,35,36]. Lipids are one of the major components of biological membranes, including the plasma membrane, the interface between the cell and the environment. Abiotic stresses like water deficit and temperature stress trigger lipid-dependent signaling cascades that activate plant adaptation processes to deal with stressful environments [37,38,39,40,41]. It was reported that *LOX* genes are involved in various growth and development processes and play important roles in plant resistance to abiotic stress, including root growth and plant development [42,43,44,45]. Based on the functions of lipid metabolism in plant abiotic stress tolerance above and the results in this research, we speculated that maybe some transcription factors or function proteins involved in cellulose metabolism, lipid synthesis and metabolism could interact with TaFBA1 to mediate the drought tolerance in wheat. Therefore, that will be the next aim to explore the underlying mechanisms in relation to the *TaFBA1*-mediated response to drought. 

### 3.3. Enhanced Root Water Absorption Capacity of TaFBA1-OE Wheat Made Contribution to Increased Drought Tolerance

Maintaining sufficient available water is crucial for plants to survive during drought stress. Water uptake and transport directly influence plant growth and development, especially under dehydration stress, and directly affect the normal metabolism of plants [46]. Therefore, plants will continuously optimize their roots to ensure their water and nutrient supplies, especially under drought stress [27,28]. For example, root volume and vitality affect nutrient and water absorption, and thus, yield [47,48]. The transmembrane transport of water is mainly mediated by aquaporins (AQPs), which regulate plant osmotic balance by controlling water transport and can interact with stress response proteins to cope with drought stress [49]. In *Arabidopsis*, Jang et al. [50] found that several aquaporin genes were significantly up-regulated or down-regulated under drought stress. In our study, there was no significant difference in the root growth status of all lines after drought treatment, but the root vitality and AQP activity were significantly higher in the OE lines than in the WT and RNAi lines (Figure 10D,E), indicating that the roots of the OE lines had strong water absorption ability and could transfer more water into plant cells to improve their drought tolerance. 

A stoma consists of a pair of guard cells that control the flow of water and gas in and out of the leaves. Stomatal aperture is extremely sensitive to environmental conditions such as light, gas, and temperature [51]. Under drought conditions, larger stomatal aperture increases transpiration and the consumption of available water in plants, which is unfavorable to water accumulation. But opened stoma contributes to the absorption of CO_2_ for photosynthesis and the accumulation of biomass [52]. Therefore, it is worthwhile to explore the balance between photosynthesis and transpiration. We found that the *TaFBA1*-OE lines had a greater proportion of fully and half-opened stomas (Figure 8A,B) and a higher water loss rate (Figure 8C) than WT. Surprisingly, the water content of the *TaFBA1*-OE leaves after drought stress was higher. Based on the analyses above, we believed that the stronger root vitality (Figure 10D) and higher AQP activity (Figure 10E) in the *TaFBA1*-OE lines supported the ability of the root system to absorb and transport water. At the same time, the higher soluble sugar content in the OE lines (Figure 10F) could maintain the osmotic potential in the leaf cells, helping them to hold on to intracellular water (Figure 8D). 

The phytohormone ABA induces stomatal closure and is considered to be an important response factor to drought stress. When suffering from drought stress, plants usually accelerate stomatal closure to hold water content for normal plant growth [4]. However, An et al. [6] reported that *TaFBA1*-overexpressing *Arabidopsis* was insensitive to ABA and that the TaFBA1 regulation on drought tolerance may be independent of ABA synthesis. Here, the wheat OE lines closed their stoma slower than the WT and RNAi lines, and the response to ABA was also similar to the study from An et al. [6]. This suggested the overexpression of *TaFBA1* decreased the sensitivity of wheat to ABA. In this respect, the physiological mechanisms through which *TaFBA1* responds to drought stress differ from many other drought response genes.

### 3.4. TaFBA1 Increased Wheat Grain Size, Especially under Drought Stress

Maintaining yield is often the long-term aim of crop studies related to abiotic stress [8]. Wheat is a globally important food crop, and years of research and breeding have led to increased stable yields [53,54]. Seed size is closely related to wheat yield and is regulated by many signaling pathways, including the UPS pathway [55]. The studies in model plants *Arabidopsis* and other food or oil crops indicated that E3 ligases play important roles in yield traits but that different genes function differently in different species [19,56]. Here, we found that under normal conditions, the seeds of OE lines were fuller than WT and the RNAi lines and the difference became more significant after drought treatment (Figure 3). The corresponding statistical results of yield traits in Figure 3 indicated that it was the filling status rather than the number of grains per spike that directly affected the yield of the OE lines. The grain weight of wheat is determined by the sink capacity and accumulation of dry matter and photosynthetic capacity and filling intensity affect substance accumulation [57,58]. Our examination of the photosynthetic capacity of all lines found that the Pn of the OE lines was significantly higher than that of the WT and RNAi lines after drought stress (Figure 4), which supported higher seed filling in the *TaFBA1*-OE lines. The molecular mechanisms underlying larger seed size in the *TaFBA1*-OE lines need to be further explored then.

## 4. Materials and Methods

### 4.1. Plant Materials and Growth Conditions

Wheat (*Triticum aestivum* L.) plants were used for physiological and molecular analyses. For germination, wheat grains were surface sterilized in 2% (*w*/*v*) sodium hypochlorite (NaClO) and then laid on moistened filter paper for 2 d at 25 °C. Seedlings of uniform size were transplanted into steam-sterilized soil mix (matrix, vermiculite, and nutrient soil, 1:1:1, *v*/*v*/*v*) and then placed into a growth chamber (light/dark temperature 25 °C/23 °C) under 300 μmol m^−2^ s^−1^ illumination and a relative humidity of 75% [59]. The wheat cultivar Shannong 16 was used to amplify cDNA sequences of *TaFBA1* [20]. The wheat cultivar CB037 was used for the generation of *TaFBA1*-OE and RNAi transgenic plants.

### 4.2. Vector Construction, Generation of Transgenic Wheat and Verification of Transgenic Lines

To generate the overexpression vector for wheat transformation, the cDNA of *TaFBA1* was amplified and cloned into the pEarleyGate101 vector after the 35S promoter [6]. To construct the RNAi vector for knocking down TaFBA1, two truncated fragments of *TaFBA1* CDS (△TaFBA1) were amplified and inserted into the pC336 vector, driven under the Ubiquitin promoter, to form an intron-containing hairpin RNA constructs (△TaFBA1-intron-△TaFBA1). The OE and RNAi constructs are shown in Figure 1A,B. The constructs were introduced into immature embryos of wheat cv. CB037 by particle bombardment as described [12]. Primers used for these amplifications are listed in Table S1. 

Verification of transgenic wheat by herbicide, PCR, and qRT-PCR analyses. For selection of the bar gene, 80 mg/L glyphosate was applied to 7-day-old wheat seedlings to initially screen transgenic T0 wheat plants. The herbicide-tolerant seedlings were then sampled for DNA extraction and PCR analysis. For qRT-PCR analysis, total RNA was extracted from the leaves of wheat seedlings using TRIZOL reagent (Vazyme, Nanjing, China). The cDNA was synthesized, and qRT-PCR was performed using a ChamQ Universal SYBR qPCR Master Mix Kit (Vazyme, Nanjing, China). The relevant primers are listed in Table S1. All reactions were run in triplicate. The confirmed transgenic lines were grown, and their progeny were screened for positive segregants. Homozygous transgenic plants were obtained in the T3 generation and used for stress-tolerant test experiments.

### 4.3. Drought Stress Treatment

Transgenic wheat seeds (T3) from two *TaFBA1*-overexpressing lines (FO3, FO5), two *TaFBA1*-RNAi lines (FR2, FR8) and their wild-type (WT; CB037) were germinated for about 3 days on moist filter paper in a culture dish (25 °C). 

For the analysis of wheat seedling phenotypes following drought stress, seeds were then put in a bottomless 96-well plate that was placed in a shallow container and incubated in a growth chamber under a 16-h light (25 °C) /8-h dark (23 °C) photoperiod in Hoagland’s nutrient solution. After growth for 3 weeks, the seedlings were at the two-leaf stage and the nutrient solution was adjusted to contain 20% (*w*/*v*) PEG6000. After 21 days of growth under osmotic stress, all lines were grown again in Hoagland’s solution for a week. 

For natural drought experiments in soil, the wheat plants were grown in a soil-containing potting mix for 2 weeks under normal conditions before drought stress was created by withholding water for 25 days. All seedlings were then re-watered. The leaf wilting rate and fresh weight of all lines were recorded. For PEG6000 treatment, 3-day-old seedlings were transferred to containers with soil mix in containers, and when grown up for 11 days, after which they were watered with 20% PEG6000 for about 5 days. The control plants were irrigated with 200 mL water every 3 days. Plants were visualized and sampled to measure some physiological parameters. Three independent experiments were performed to obtain data for statistical analyses [4,53].

For drought stress treatment of wheat at the heading stage, six germinated seeds were grown in the same pot (matrix, vermiculite, and nutrient soil (1:1:3, *v*/*v*/*v*), 4 L, 6 plants per pot) under imitated natural conditions (25 °C daytime and 23 °C nighttime temperatures). Water was replaced by 20% PEG6000 from 32-day-old plants for 14 days and 21 days, while the control plants were irrigated with 400 mL water every 3 days [29,53]. Plants were photographed and relative physiological and photosynthetic parameters were monitored as reported [60]. Agronomic traits including hundred grain weight, grain number per spike, grain length and grain width were measured in the harvested plants. 

For analysis of root morphology following drought stress, one-leaf seedlings were cultured in hydroponics for 4 days in 20% PEG6000 solution. The roots phenotype was photographed before and after exposure to the PEG, and root length and shoot length of all lines were recorded. Root physiological parameters, such as root vitality and AQP activity, were measured for lines grown under normal and drought conditions. The AQP activity was assessed by enzyme-linked immunosorbent assay (ELISA). Fresh root tips (0.5 g, 2 cm) were used to measure root activity by the 2,3,5-triphenyltetrazolium chloride (TTC) method [24].

### 4.4. Chlorophyll Content and Photosynthetic Parameter Assays

The chlorophyll content was measured using a UV spectrophotometric method as described previously [60]. The photosynthetic gas exchange parameters of the flag leaves and ΦPSII, Fv/Fm were measured using the procedures described by Wang et al. [60]. 

### 4.5. Malondialdehyde Content, Electrolyte Leakage, Proline and Soluble Sugar Contents 

Two-week-old plants were treated with 20% PEG6000 for about 5 days. The MDA content was measured in accordance with the described method by Zhou et al. [20]. Electrolyte leakage was measured according to the literature [61]. Proline content was determined according to previously described methods [13]. The soluble sugar content was determined using the anthrone method [62].

### 4.6. Antioxidant Analysis Experiments

Two-week-old seedlings grown in soil mix were watered with 20% PEG6000 for about 5 days. The leaves from normal growth and dehydration conditions were collected to conduct ROS analysis and antioxidative enzyme activities assays. DAB and NBT staining, as well as H_2_O_2_ concentration and O^2−^ production were conducted as described previously [61]. The activities of antioxidant enzymes, including CAT, POD, SOD, APX, MDAR, DHAR, GR, and GPx, were measured as described by the literature [59].

Wheat seedlings grown in soil mix under-watered conditions for 2 weeks were sprayed with 10 μM MV. Phenotypes of wheat seedlings before and 14 days after spraying with 10 μM MV were photographed and the leaf wilting rate was recorded.

Two-week-old transgenic wheat and WT plants were subjected to water and 20% PEG6000 solution for 5 days. The leaves of these plants were then used for the immunological analysis of protein carbonylation. Briefly, total protein was extracted from the wheat leaves followed by the determination of protein concentration according to Bradford [63]. The separation of protein by SDS-PAGE and detection of carbonylation level were carried out according to the description [13].

### 4.7. Stomatal Aperture

To analyze the stomatal aperture of WT and transgenic wheat leaves, seedlings were exposed to 20% PEG6000 or water for 5 days. The middle part of the nether leaf epidermis was covered with transparent nail polish and allowed to dry for about 3 min. The dried nail polish was peeled off, removing a layer of epidermis, and placed on a glass slide for microscopic observation. The aperture status stoma was observed and imaged under an AXIO microscope (Zeiss, Jena, Germany) at 10 × 40 amplification [64]. Stomatal types were defined and analyzed according to the method [65].

### 4.8. Seed Germination and Seedling Growth Assays

To assay the response of seed germination to ABA, wheat seeds from two *TaFBA1*-overexpressing lines (FO3, FO5), two TaFBA1-RNAi lines (FR2, FR8), and WT (CB037) were sterilized using 70% ethanol for 2 min and then disinfected with 4% sodium hypochlorite (NaClO) for 10 min. All sterilized seeds were rinsed in sterilized deionized water at least five times and germinated on 1/2 Murashige-Skoog (MS) medium containing different concentrations of ABA (0, 1, 6, 15 μM) under a 16-h light/8-h dark photoperiod at 25 °C. The root length and shoot length were measured after they grew on the different 1/2 MS media for 5 days.

### 4.9. Water Loss Rate, Relative Water Content

To examine water loss in the WT and transgenic lines, the leaves of 6 plants from each line grown under either control or drought stress conditions were harvested. The leaves were retained at room temperature and weighed at 0, 0.5, 1, 1.5, 2, 2.5, 3, 4, 5, 6 and 9 h. Water loss was represented as the proportion of fresh weight lost (calculated using the initial weight of the plant samples) over time [29,66]. Relative water content was determined using the following formula: RWC = (fresh weight − dry weight)/(rehydrated weight − dry weight) [20]. 

### 4.10. RNA-Seq Assay

Two-week-old CB037 and TaFBA1-overexpressing wheat plants were maintained in a well-watered state or subjected to drought for 6 h before leaves were collected for RNA extraction. RNA-seq was performed using an Illumina HiseqTM 2500/4000 by Gene Denovo Biotechnology Co., Ltd. (Guangzhou, China) with three biological replicates per sample. Bioinformatic analysis was performed using Omicsmart, a real-time interactive online platform for data analysis (http://www.omicsmart.com). DEGs were identified using the method described in Kan et al. [67]. DEGs were identified as genes with a fold change ≥2 and a false discovery rate (FDR) < 0.05 by comparison. GO term enrichment analysis of the gene sets of interest was performed as described [68]. The same samples were used for the validation of transcription data via qRT-PCR.

### 4.11. qRT-PCR Assay

Samples were collected from wheat seedlings and leaves treated or not treated with PEG6000 for RNA extraction and reverse transcription using HiScript Q RT SuperMix for qPCR (Vazyme, Nanjing, China) according to the manufacturer’s instructions. qRT-PCR was performed using the AceQ qPCR SYBR Green Master Mix (Vazyme, Nanjing, China) and the CFX96 Real-Time System (Bio-Rad, Hercules, CA, USA). All primers used for qRT-PCR experiments are listed in Table S2. 

### 4.12. Statistical Analysis

All experiments were performed in at least three independent biological replicates. Data points represent mean ± SD (standard deviation) of three replicates. The differences between non-transformed control and transformed plants were analyzed using Student’s *t*-test, and asterisks above columns in the figures indicated statistical differences (* *p* < 0.05; ** *p* < 0.01).

## 5. Conclusions

According to the results in this paper, we speculate that *TaFBA1* regulates multiple systems that support wheat tolerance to drought stress. On the one hand, TaFBA1 enhanced the antioxidant capacity of transgenic wheat by inducing the expression of genes related to the antioxidant system to reduce cellular ROS accumulation which would reduce the oxidative damage to the chloroplast, and, as a result, improve the drought tolerance of OE lines. On the other hand, *TaFBA1* negatively regulated wheat sensitivity to ABA, resulting in slower stomatal closure and increased water loss under drought stress, but this was negated by the stronger water absorption capacity of the roots which allowed the cells to maintain a higher water content despite the increased transpiration. Meanwhile, the larger stomatal aperture helped the plant leaves to absorb more CO_2_, reduced the production of excess reducing power and ensured higher carbon assimilation. 

Together, the results showed that TaFBA1 played a positive role in drought tolerance in wheat. RNA-seq analysis showed that lipid metabolism and fatty acid synthesis genes, as well as genes from cellulose synthesis, might also play important roles in the *TaFBA1*-regulated wheat tolerance to drought. Our next exploration of the mechanisms underlying drought resistance in this important crop species is promising and we hope to impact variety breeding with our ongoing discoveries.

## Data Availability

The data is contained within the manuscript and Supplementary Materials.

## Supplementary Materials

The following supporting information can be downloaded at: https://www.mdpi.com/article/10.3390/plants13182588/s1, Figure S1. E3 ligase activity of transgenic wheat lines. Figure S2. Analysis of drought tolerance in WT ‘CB037’and transgenic wheat lines at the seedling stage. Figure S3. Effects of drought stress on protein carbonylation levels in WT and transgenic wheat lines. Figure S4. Effects of methyl viologen (MV) treatment on WT and transgenic wheat lines. Figure S5. The response of *TaFBA1* to 20% PEG6000 in the leaves of CB037. Table S1. The primers used for PCR amplification. Table S2. The primers used for qRT-PCR.

## References

[1] Langridge P., Reynolds M. (2021). Breeding for drought and heat tolerance in wheat. Theor. Appl. Genet..

[2] Ma J.H., Geng Y.K., Liu H., Zhang M.Q., Liu S.J., Hao C.Y., Hou J., Zhang Y.F., Zhang D.J., Zhang W.J. (2023). TaTIP41 and TaTAP46 positively regulate drought tolerance in wheat by inhibiting PP2A activity. J. Integr. Plant Biol..

[3] Zhang L.L., Zheng Y., Xiong X.X., Li H., Zhang X., Song Y.L., Zhang X.H., Min D.H. (2023). The wheat VQ motif-containing protein TaVQ4-D positively regulates drought tolerance in transgenic plants. J. Exp. Bot..

[4] Cui L.G., Shan J.X., Shi M., Gao J.P., Lin H.X. (2015). DCA1 Acts as a Transcriptional Co-activator of DST and Contributes to Drought and Salt Tolerance in Rice. PLoS Genet..

[5] Vishwakarma K., Upadhyay N., Kumar N., Yadav G., Singh J., Mishra R.K., Kumar V., Verma R., Upadhyay R.G., Pandey M. (2017). Abscisic acid signaling and abiotic stress tolerance in plants: A review on current knowledge and future prospects. Front. Plant Sci..

[6] An J., Li Q.X., Yang J.J., Zhang G.Q., Zhao Z.X., Wu Y.Z., Wang Y., Wang W. (2019). Wheat F-box Protein TaFBA1 Positively Regulates Plant Drought Tolerance but Negatively Regulates Stomatal Closure. Front. Plant Sci..

[7] Qiu D., Hu W., Zhou Y., Xiao J., Hu R., Wei Q.H., Zhang Y., Feng J.L., Sun F.S., Sun J.T. (2021). TaASR1-D confers abiotic stress resistance by affecting ROS accumulation and ABA signalling in transgenic wheat. Plant Biotech. J..

[8] Mao H.D., Jian C., Cheng X.X., Chen B., Mei F.M., Li F.F., Zhang Y.F., Li S.M., Du L.Y., Li T. (2022). The wheat ABA receptor gene *TaPYL1-1B* contributes to drought tolerance and grain yield by increasing water-use efficiency. Plant Biotechnol. J..

[9] Mei F.M., Chen B., Du L.Y., Li S.M., Zhu D.H., Chen N., Zhang Y.F., Li F.F., Wang Z.X., Cheng X.X. (2022). A gain-of-function allele of a DREB transcription factor gene ameliorates drought tolerance in wheat. Plant Cell.

[10] Mao H.D., Li S.M., Chen B., Jian C., Mei F.M., Zhang Y.F., Li F.F., Chen N., Li T., Du L.Y. (2022). Variation in cis-regulation of a NAC transcription factor contributes to drought tolerance in wheat. Mol. Plant.

[11] Finley D., Ulrich H.D., Sommer T., Kaiser P. (2012). The ubiquitin–proteasome system of saccharomyces cerevisiae. Genetics.

[12] Wang W.L., Wang W.Q., Wu Y.Z., Li Q.X., Zhang G.Q., Shi R.R., Yang J.J., Wang Y., Wang W. (2020). The involvement of wheat U-box E3 ubiquitin ligase TaPUB1 in salt stress tolerance. J. Integr. Plant Biol..

[13] Li Q.X., Wang W.Q., Wang W.L., Zhang G.Q., Liu Y., Wang Y., Wang W. (2018). Wheat F-Box protein gene *TaFBA1* is involved in plant tolerance to heat stress. Front. Plant Sci..

[14] Kim Y.Y., Cui M.H., Noh M.S., Jung K.W., Shin J.S. (2017). The FBA motif-containing protein AFBA1 acts as a novel positive regulator of ABA response in *Arabidopsis*. Plant Cell Physiol..

[15] Jia F.J., Wang C.Y., Huang J.G., Yang G.D., Wu C.A., Zheng C.C. (2015). SCF E3 ligase PP2-B11 plays a positive role in response to salt stress in *Arabidopsis*. J. Exp. Bot..

[16] Sharma E., Bhatnagar A., Bhaskar A., Majee S.M., Kieffer M., Kepinski S., Khurana P., Khurana J.P. (2023). Stress-induced F-Box protein-coding gene *OsFBX257* modulates drought stress adaptations and ABA responses in rice. Plant Cell Environ..

[17] Li Z.S., Wang X.Y., Cao X.C., Chen B.Z., Ma C.K., Lv J.Y., Sun Z.M., Qiao K.K., Zhu L.F., Zhang C.J. (2021). GhTULP34, a member of tubby-like proteins, interacts with GhSKP1A to negatively regulate plant osmotic stress. Genomics.

[18] Zhang Y.E., Xu W., Li Z., Deng X.W., Wu W., Xue Y. (2008). F-box protein DOR functions as a novel inhibitory factor for abscisic acid-induced stomatal closure under drought stress in *Arabidopsis*. Plant Physiol..

[19] Chen Y., Xu Y.Y., Luo W., Li W.X., Chen N., Zhang D.J., Chong K. (2013). The F-Box Protein OsFBK12 Targets OsSAMS1 for Degradation and Affects Pleiotropic Phenotypes, Including Leaf Senescence, in Rice. Plant Physiol..

[20] Zhou S., Sun X., Yin S. (2014). The role of the F-box gene *TaFBA1* from wheat (*Triticum aestivum* L.) in drought tolerance. Plant Physiol. Biochem..

[21] Al-Saharin R., Hellmann H., Mooney S. (2022). Plant E3 Ligases and Their Role in Abiotic Stress Response. Cells.

[22] Li B.W., Gao S., Yang Z.M., Song J.B. (2022). The F-box E3 ubiquitin ligase AtSDR is involved in salt and drought stress responses in *Arabidopsis*. Gene.

[23] Kong X.Z., Zhou S.M., Yin S.H., Zhao Z.X., Han Y.Y., Wang W. (2016). Stress-Inducible Expression of an F-box Gene *TaFBA1* from Wheat Enhanced the Drought Tolerance in Transgenic Tobacco Plants without Impacting Growth and Development. Front. Plant Sci..

[24] Zhao Z.X., Zhang G.Q., Zhou S.M., Ren Y.Q., Wang W. (2017). The improvement of salt tolerance in transgenic tobacco by overexpression of wheat F-box gene *TaFBA1*. Plant Sci..

[25] Chaves M.M., Flexas J., Pinheiro C. (2009). Photosynthesis under drought and salt stress: Regulation mechanisms from whole plant to cell. Ann. Bot..

[26] Xu N.N., Chu Y.L., Chen H.L., Li X.X., Wu Q., Jin L., Wang G.X., Huang J.L. (2018). Rice transcription factor OsMADS25 modulates root growth and confers salinity tolerance via the ABA-mediated regulatory pathway and ROS scavenging. PLoS Genet..

[27] Yu H., Chen X., Hong Y.Y., Wang Y., Xu P., Ke S.D., Liu H.Y., Zhu J.K., Oliver D.J., Xiang C.B. (2008). Activated expression of an *Arabidopsis* HD-START protein confers drought tolerance with improved root system and reduced stomatal density. Plant Cell.

[28] Ma H.Z., Liu C., Li Z.X., Ran Q.J., Xie G.N., Wang B.M., Fang S., Chu J.F., Zhang J.R. (2018). ZmbZIP4 contributes to stress resistance in maize by regulating ABA synthesis and root development. Plant Physiol..

[29] Yang J.J., Zhang G.Q., An J., Li Q.X., Chen Y.H., Zhao X.Y., Wu J.J., Wang Y., Hao Q.Q., Wang W.Q. (2020). Expansin gene *TaEXPA2* positively regulates drought tolerance in transgenic wheat (*Triticum aestivum* L.). Plant Sci..

[30] Ding S., Zhang B., Qin F. (2015). *Arabidopsis* RZFP34/CHYR1, a ubiquitin E3 ligase, regulates stomatal movement and drought tolerance via SnRK2.6-mediated phosphorylation. Plant Cell.

[31] Guo C.K., Yao L.Y., You C.J., Wang S.S., Cui J., Ge X.C., Ma H. (2016). MID1 plays an important role in response to drought stress during reproductive development. Plant J..

[32] Kaouthar F., Ameny F.K., Yosra K., Walid S., Ali G., Faiçal B. (2016). Responses of transgenic *Arabidopsis* plants and recombinant yeast cells expressing a novel durum wheat manganese superoxide dismutase TdMnSOD to various abiotic stresses. J. Plant Physiol..

[33] Shinozaki K., Yamaguchi-Shinozaki K. (2007). Gene networks involved in drought stress response and tolerance. J. Exp. Bot..

[34] Endler A., Kesten C., Schneider R., Zhang Y., Ivakov A., Froehlich A., Funke N., Persson S. (2015). A mechanism for sustained cellulose synthesis during salt stress. Cell.

[35] Wang T., McFarlane H.E., Persson S. (2016). The impact of abiotic factors on cellulose synthesis. J. Exp. Bot..

[36] Lou H.Y., Tucker M.R., Shirley N.J., Lahnstein J., Yang X.J., Ma C., Schwerdt J., Fusi R., Burton R.A., Band L.R. (2022). The *cellulose synthase-like F3* (*CslF3*) gene mediates cell wall polysaccharide synthesis and affects root growth and differentiation in barley. Plant J..

[37] Hou Q.C., Ufer G., Bartels D. (2016). Lipid signalling in plant responses to abiotic stress. Plant Cell Environ..

[38] He M., He C.Q., Ding N.Z. (2018). Abiotic stresses: General defenses of land plants and chances for engineering multistress tolerance. Front. Plant Sci..

[39] He M., Ding N.Z. (2020). Plant Unsaturated Fatty Acids: Multiple Roles in Stress Response. Front. Plant Sci..

[40] Zhang M., Barg R., Yin M.G., Gueta-Dahan Y., Leikin-Frenkel A., Salts Y., Shabtai S., Ben-Hayyim G. (2005). Modulated fatty acid desaturation via overexpression of two distinct ω-3 desaturases differentially alters tolerance to various abiotic stresses in transgenic tobacco cells and plants. Plant J..

[41] Fan J.L., Yu L.H., Xu C.C. (2017). A central role for triacylglycerol in membrane lipid breakdown, fatty acid β-oxidation, and plant survival under extended darkness. Plant Physiol..

[42] Wozniak A., Kesy J., Glazinska P., Glinkowski W., Narozna D., Bocianowski J., Rucinska-Sobkowiak R., Mai V.C., Krzesinski W., Samardakiewicz S. (2023). The Influence of Lead and Acyrthosiphon pisum (Harris) on Generation of *Pisum sativum* Defense Signaling Molecules and Expression of Genes Involved in Their Biosynthesis. Int. J. Mol. Sci..

[43] López M.A., Vicente J., Kulasekaran S., Vellosillo T., Martínez M., Irigoyen M.L., Cascón T., Bannenberg G., Hamberg M., Castresana C. (2011). Antagonistic role of 9-lipoxygenase-derived oxylipins and ethylene in the control of oxidative stress, lipid peroxidation and plant defence. Plant J..

[44] Upadhyay R.K., Handa A.K., Mattoo A.K. (2019). Transcript abundance patterns of 9-and 13-lipoxygenase subfamily gene members in response to abiotic stresses (heat, cold, drought or salt) in tomato (*Solanum lycopersicum* L.) highlights member-specific dynamics relevant to each stress. Genes.

[45] Sun Q., Zhang B., Yang C.L., Wang W.L., Xiang L., Wang Y.P., Chan Z.L. (2022). Jasmonic acid biosynthetic genes *TgLOX4* and *TgLOX5* are involved in daughter bulb development in tulip (*Tulipa gesneriana*). Hortic. Res..

[46] Mega R., Abe F., Kim J.S., Tsuboi Y., Tanaka K., Kobayashi H., Sakata Y., Hanada K., Tsujimoto H., Kikuchi J. (2019). Tuning water-use efficiency and drought tolerance in wheat using abscisic acid receptors. Nat. Plants.

[47] Khandal H., Gupta S.K., Dwivedi V., Mandal D., Sharma N.K., Vishwakarma N.K., Pal L., Choudhary M., Francis A., Malakar P. (2020). Root-specific expression of chickpea cytokinin oxidase/ dehydrogenase 6 leads to enhanced root growth, drought tolerance and yield without compromising nodulation. Plant Biotechnol. J..

[48] Zhao S., Gao H.B., Jia X.M., Li X.W., Mao K., Ma F.W. (2021). The γ-clade HD-Zip I transcription factor MdHB-7 regulates salt tolerance in transgenic apple (*Malus domestica*). Plant Soil.

[49] Aroca R., Porcel R., Ruiz-Lozano J.M. (2012). Regulation of root water uptake under abiotic stress conditions. J. Exp. Bot..

[50] Jang J.Y., Kim D.J., Kim Y.O., Kim J.S., Kang H. (2004). An expression analysis of a gene family encoding plasma membrane aquaporins in response to abiotic stresses in *Arabidopsis thaliana*. Plant Mol. Biol..

[51] Sugiyama Y., Uraji M., Watanabe-Sugimoto M., Okuma E., Munemasa S., Shimoishi Y. (2012). FIA functions as an early signal component of abscisic acid signal cascade in Vicia faba guard cells. J. Exp. Bot..

[52] Saradadevi R., Bramley H., Palta J.A., Siddique K.H.M. (2017). Stomatal behaviour under terminal drought affects post-anthesis water use in wheat. Funct. Plant Biol..

[53] Zhang N., Yin Y.J., Liu X.Y., Tong S.M., Xing J.W., Zhang Y., Pudake R.N., Izquierdo E.M., Peng H.R., Xin M.M. (2017). The E3 ligase TaSAP5 alters drought stress responses by promoting the degradation of DRIP proteins. Plant Physiol..

[54] Li S.N., Lin D.X., Zhang Y.W., Deng M., Chen Y.X., Lv B., Li B.S., Lei Y., Wang Y.P., Zhao L. (2022). Genome-edited powdery mildew resistance in wheat without growth penalties. Nature.

[55] Li N., Li Y. (2016). Signaling pathways of seed size control in plants. Curr. Opin. Plant Biol..

[56] Miller C., Wells R., McKenzie N., Trick M., Ball J., Fatihi A., Dubreucq B., Chardot T., Lepiniec L., Bevan M.W. (2019). Variation in expression of the HECT E3 ligase UPL3 modulates LEC2 levels, seed size, and crop yields in *Brassica napus*. Plant Cell.

[57] Liu L.C., Tong H.G., Xiao Y.H., Che R.H., Xu F., Hu B., Liang C., Chu J., Li J., Chu C. (2015). Activation of Big Grain1 significantly improves grain size by regulating auxin transport in rice. Proc. Natl. Acad. Sci. USA.

[58] Brinton J., Simmonds J., Minter F., Leverington-Waite M., Snape J., Uauy C. (2017). Increased pericarp cell length underlies a major quantitative trait locus for grain weight in hexaploid wheat. New Phytol..

[59] Liu S.T., Liu S.W., Wang M., Wei T.D., Meng C., Wang M., Xia G.M. (2014). A Wheat *SIMILAR To RCD-ONE* Gene Enhances Seedling Growth and Abiotic Stress Resistance by Modulating Redox Homeostasis and Maintaining Genomic Integrity. Plant Cell.

[60] Wang W.Q., Li Q.X., Tian F.X., Deng Y.M., Wang W.L., Wu Y.Z., Yang J.J., Wang Y., Hao Q.Q., Wang W. (2019). Wheat NILs contrasting in grain size show different expansin expression, carbohydrate and nitrogen metabolism that are correlated with grain yield. Field Crop. Res..

[61] Ma Y.M., Yang C., He Y., Tian Z.H., Li J.X. (2017). Rice OVATE family protein 6 regulates plant development and confers resistance to drought and cold stresses. J. Exp. Bot..

[62] Wang N., Zhang W.X., Qin M.Y., Li S., Qiao M., Liu Z.H., Xiang F.N. (2017). Drought tolerance conferred in Soybean (*Glycine max*. L) by GmMYB84, a novel R2R3-MYB transcription factor. Plant Cell Physiol..

[63] Bradford M.M. (1976). A rapid and sensitive method for the quantitation of microgram quantities of protein utilizing the principle of protein-dye binding. Anal. Biochem..

[64] Huang L.C., Chen L., Wang L., Yang Y.L., Rao Y.C., Ren D.Y., Dai L.P., Gao Y.H., Zou W.W., Lu X.L. (2019). A Nck-associated protein 1-like protein affects drought sensitivity by its involvement in leaf epidermal development and stomatal closure in rice. Plant J..

[65] Matsuda S., Takano S., Sato M., Furukawa K., Nagasawa H., Yoshikawa S., Kasuga J., Tokuji Y., Yazaki K., Nakazono M. (2016). Rice stomatal closure requires guard cell plasma membrane ATP-binding cassette transporter RCN1/OsABCG5. Mol. Plant.

[66] Wang Z.Y., Tian X.J., Zhao Q.Z., Liu Z.Q., Li X.F., Ren Y.K., Tang J.Q., Fang J., Xu Q.J., Bu Q.Y. (2018). The E3 ligase DROUGHT HYPERSENSITIVE negatively regulates cuticular wax biosynthesis by promoting the degradation of transcription factor ROC4 in rice. Plant Cell.

[67] Kan L., Liao Q.C., Chen Z.P., Wang S.Y., Ma Y.F., Su Z.Y., Zhang L. (2021). Dynamic Transcriptomic and Metabolomic Analyses of *Madhuca pasquieri* (Dubard) H. J. Lam During the Post-germination Stages. Front. Plant Sci..

[68] Young M.D., Wakefield M.J., Smyth G.K., Oshlack A. (2010). 2010. Gene ontology analysis for RNA-seq: Accounting for selection bias. Genome Biol..

