# Supplementary material for: The F-Box Protein TaFBA1 Positively Regulates Drought Resistance and Yield Traits in Wheat
Source: Plants (Basel). 2024 Sep 16;13(18):2588. doi: 10.3390/plants13182588 (PMC11434774; doi:10.3390/plants13182588)
Supplement: Supplementary file 1 [file plants-13-02588-s001.zip › plants-3140445-supplementary.pdf]

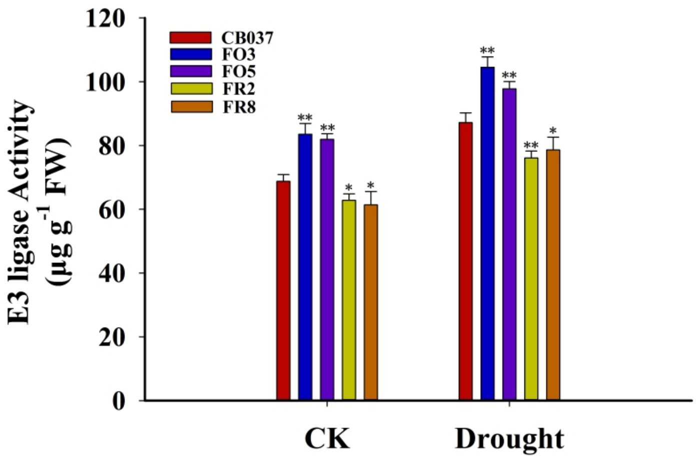

Figure S1. E3 ligase activity of transgenic wheat lines.

2-week-old wheat grown under normal condition were exposed to 20% PEG6000, and the plant leaves of CK and drought conditions were sampled to extract total protein. The E3 ligase activities of CB037 (WT) and two OE lines and two RNA interference lines (in the T3 generation) were determined by the Double Antibody Sandwich Method.

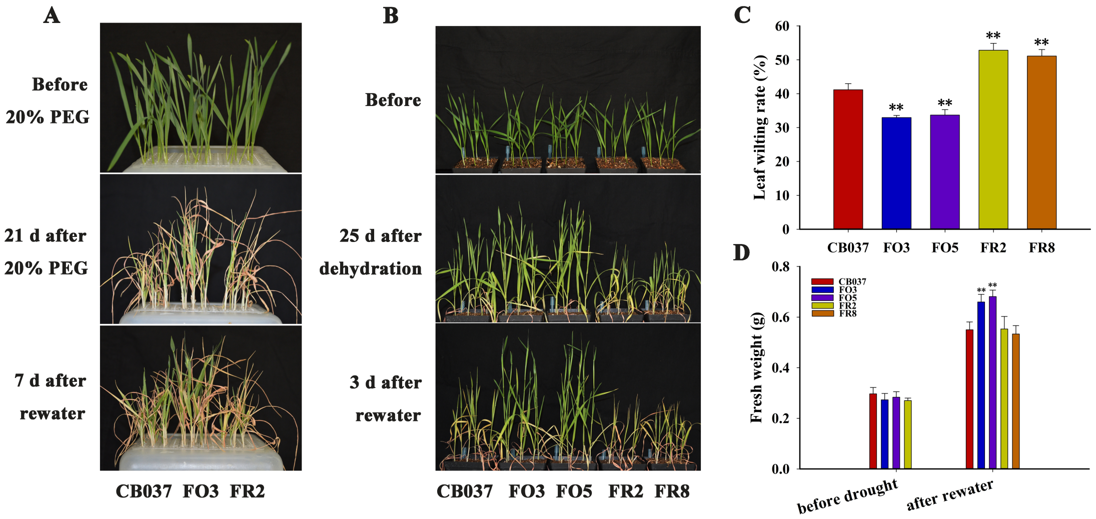

Figure. S2. Analysis of drought tolerance in WT 'CB037' and transgenic wheat lines at the seedling stage.

(A) Phenotype of WT and transgenic wheat seedlings at the two-leaf stage before and after treatment with 20% PEG6000 for 21 days and after re-watering for 7 days. (B) Phenotype of 2-week-old wheat seedlings before and after withholding water for 25 days and re-watering for 3 days. (C) Leaf wilting rate (%) after re-watering for 3 days. (D) Fresh weight before and after re-watering for 3 days. The data represent the mean ± SE of three biological replicates. *P <0.05; **P< 0.01.

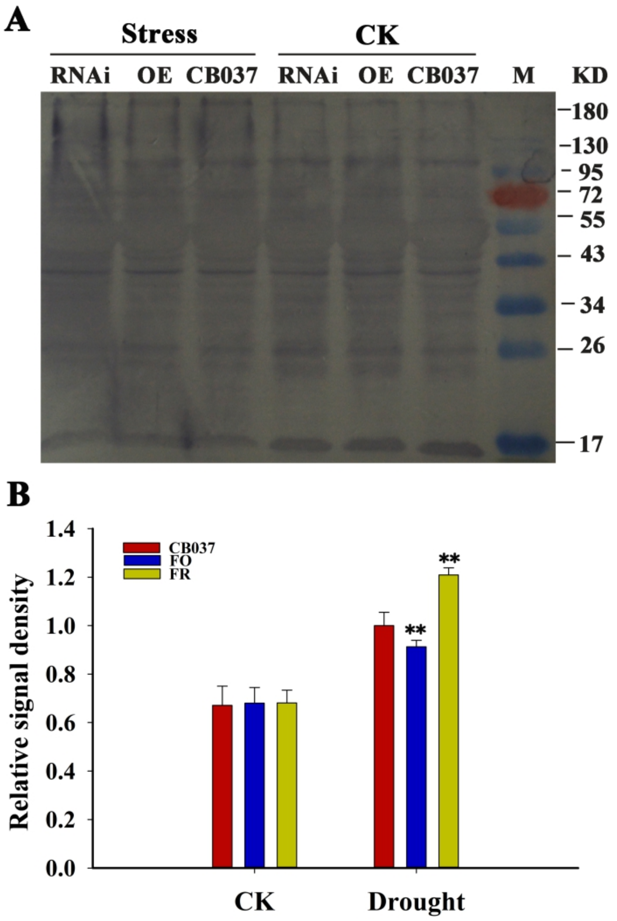

Figure. S3 Effects of drought stress on protein carbonylation levels in WT and transgenic wheat lines.

(A) Protein carbonylation levels and (B) relative signal density of carbonylated proteins in all lines grown under normal and drought stress conditions for 5 days. The data represent the mean ± SE of three biological replicates. *P <0.05; **P < 0.01.

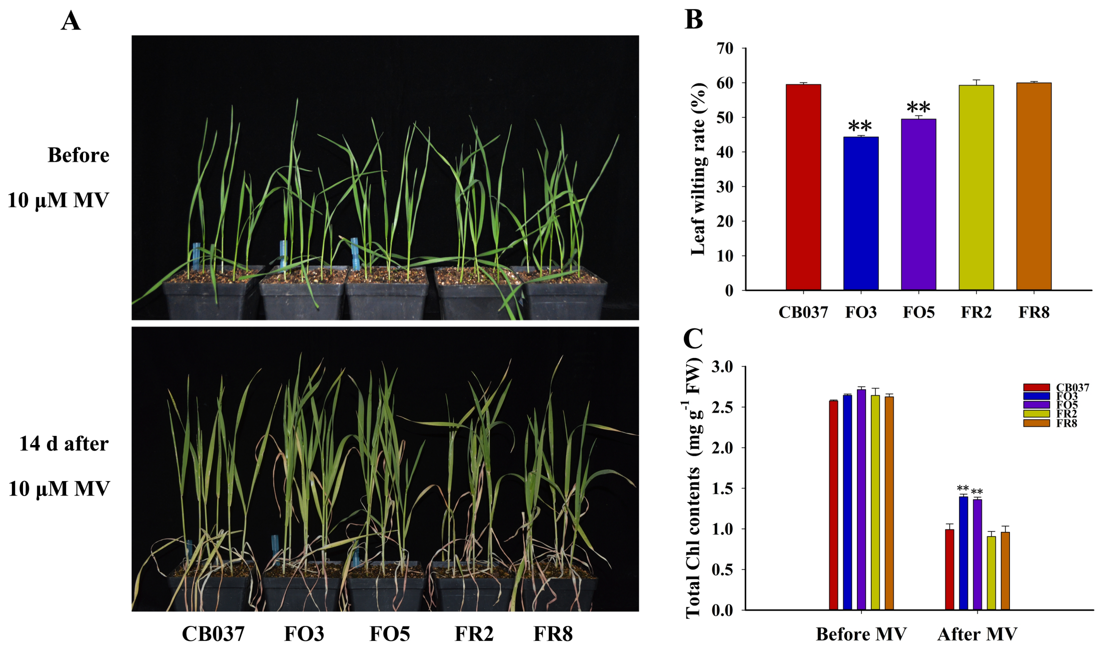

Figure. S4 Effects of methyl viologen (MV) treatment on WT and transgenic wheat lines.

(A) Phenotypes of wheat seedlings before and 14 days after spraying with 10 µM MV. (B) Leaf wilting rate (%) of all the lines after 10 µM MV treatment. (C) Total Chl contents of wheat leaves before and after MV treatment. The data represent the mean ± SE of three biological replicates. *P <0.05; **P < 0.01.

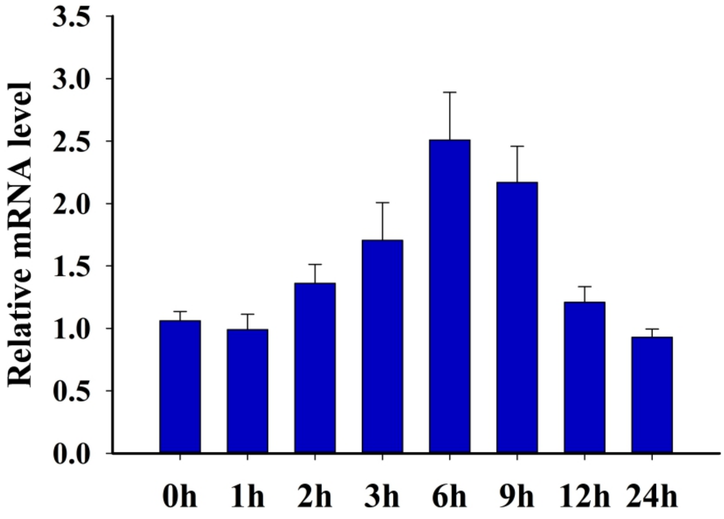

Figure. S5 The response of *TaFBA1* to 20% PEG6000 in the leaves of CB037.

Table S1. The primers used for PCR amplification.

Primer	Sequence (5 ' to 3')
TaFBA1-F	CACCGGAGCAGAGATGGAAGAGCA
TaFBA1-R	AGTCGCTGATCTCGCTCCTC
TaFBA1-RNAi-F	CACCAACAGCTTCCTGGAGGAG
TaFBA1-RNAi-R	GGCGTGGCCGTCGGCCCA
35S	CTATCCTTCGCAAGACCCTTC
LB046	TTTAGCCCTGCCTTCATACG

Table S2. The primers used for qRT-PCR.

Primer	Sequence (5 ' to 3')
Actin-F	ACCTTCAGTTGCCCAGCAAT
Actin-R	CAGAGTCGAGCACAATACCAGTTG
Tubulin-F	ATCTGTGCCTTGACCGTATCAGG
Tubulin-R	GACATCAACATTCAGGACACCATC
TaFBA1-F	AGCAGCAGAACAAGCCTGACCA
TaFBA1-R	ACGTGACGTTGGACAGCCTTTG
TaMnSOD-F	CGTCGCCAACTACAACAAGG
TaMnSOD-R	ACCACCACCCTCGCTAATG
TaCu/Zn-SOD-F	TGGGAGAGCGTTTGTTGTTC
TaCu/Zn-SOD-R	GTCTTCCACCAGCATTTCCA
TaFe-SOD-F	CCTACTGGATGAGACGGAGAG
TaFe-SOD-R	GGACGAGGACAACGACGAA
TaCAT-F	CCATGAGATCAAGGCCATCT
TaCAT-R	ATCTTACATGCTCGGCTTGG

TaPOD-F	ACCTCTTCACCAACGACATCAC
TaPOD-R	ATGGACACGCCGAACTGCT
TaAPX-F	TTGAGCCTATTAAAGCGAAGCA
TaAPX-R	ACGGGGACAAACTGACGAA
TaP5CS-F	AATAGAGGCCATGGCTGATG
TaP5CS-R	AGGGGAAGTGACCTGATCCT
TaDHAR-F	ATAATGCGAGCGAGCCTTGA
TaDHAR-R	CGCAGCTCCATTATTCATTCACA
TaGR-F	GGTTGCACTGATGGAAGGTG
TaGR-R	AATGGGTGGTTGGGAGAAAA
TaMDAR-F	TCCACCATACGAGCGACC
TaMDAR-R	ACCAGTAGAAATGATAAGTGACCC
TaLEA7-F	GTCGAGAGCAAGGACCAGAC
TaLEA7-R	CCTGTCCTGCGTGTACTGC
TaDREB6-F	GTTCCCCATCACATACTGGTCTC
TaDREB6-R	TGCTTGTGTCTGGTGCCTCT
TaFER-5B-F	*TCACTCCCTCTTCGCCTACTTC*
TaFER-5B-R	TCCTCGTCGCTTGATTCCTT
TaRD29B-F	CAAGTCGACGTGAGCAAAGA
TaRD29B-R	GCCGTATTCTTGAGCCTGTC
TaSAPK2-F	TGCTCATCGGGTCGTATCCA
TaSAPK2-R	GGCAGGCTCTTCAGGTACCA
TaGPX-F	CCCCCTGTACAAGTTCCTGA
TaGPX-R	GTCAACAACGTGACCCTCCT
TaLOX-F	CTGCAAAGCATCATTGGAGTGTCC
TaLOX-R	TGATGTCCACTTTGGCTCATCACG

TaLOX1.1-F	CCCATCGTCTTCGTCGCAAATTC
TaLOX1.1-R	TTGGCAGGTACGTGTCGTTG
TaEXPA2-F	GCCTACCTCCAGATCGGAATCTAC
TaEXPA2-R	CACCAGGTTGAAGTAGGAGTGC
TaFAD7-F	GAGGATCACTATGTTAGCGACACC
TaFAD7-R	AGCTCTGTCCAGAAGTACTCGTC
TaCSL3-2-F	GTGCACTGGGTCTGCTATTCAACC
TaCSL3-2-R	ATCGCCAATGCAAATGGGTAAAGG
TaCSL3-4-F	GCTGGCGCTCCATGTATGTTAC
TaCSL3-4-R	ACCAGACCATCGCACTATTTGGC
